# Supplementary material for: Effects of relaxation interventions during pregnancy on maternal mental health, and pregnancy and newborn outcomes: A systematic review and meta-analysis
Source: PLoS One. 2024 Jan 25;19(1):e0278432. doi: 10.1371/journal.pone.0278432 (PMC10810490; doi:10.1371/journal.pone.0278432)
Supplement: S1 Table — (DOCX) [file pone.0278432.s001.docx]

| **Supporting information table 1: Risk of bias assessment result for the included studies in the review based on the Cochrane Collaboration’s risk of bias assessment tool** | | | | | | | |
| --- | --- | --- | --- | --- | --- | --- | --- |
| Studies | Selection Bias: Random Sequence generation | Selection Bias: Allocation Concealment | Performance Bias: Blinding of participants and personnel | Detection Bias: Blinding of outcome assessment | Attrition Bias: Incomplete outcome data | Reporting Bias: Selective outcome reporting | Other Bias: Other sources  of bias |
| Bastani F, et al.2005 | Low | Low | Unclear | Unclear | Low | Unclear | Low |
| Bastani F, eta al., 2006 | Low | Low | Unclear | Unclear | Low | Unclear | Low |
| Chuntharapat S, et al. 2008 | low | Low | Unclear | Unclear | Low | High | Low |
| Chang MY, et al. , 2008 | Low | Low | Unclear | Unclear | Low | Unclear | Low |
| Satyapriy M, et al. 2009 | Low | Unclear | High | Low | Low | Unclear | Low |
| Yang M, et al.2009 | Low | Low | Unclear | Unclear | Low | Unclear | Low |
| Urech C, et al. 2010 | Low | low | Unclear | Unclear | High | Unclear | Low |
| Liu YH, et al. 2010 | Low | Unclear | High | Low | Low | Unclear | Low |
| Simavli S, et al. 2014 | Low | Low | Unclear | Unclear | Low | Unclear | Low |
| Simavli S, et al. 2014 | Low | Low | Unclear | Unclear | Low | Unclear | Low |
| Tragea C, et al. 2014 | Low | Low | Unclear | Unclear | Low | Unclear | Low |
| Guardinoa CM, et al. 2014 | Low | Unclear | Unclear | Unclear | Low | Unclear | Low |
| Newham J, et al. 2014 | Low | Low | Unclear | Unclear | Low | Unclear | Low |
| Davis K, et al. 2015 | Low | Low | Unclear | Unclear | Low | Unclear | Low |
| Chang HC, et al. 2015 | Unclear | Unclear | Unclear | Unclear | Low | Unclear | Low |
| Liu YH, et al. 2016 | Low | Unclear | Unclear | Unclear | Low | Unclear | Low |
| MuthukriShnan M, et al. 2016 | Low | Unclear | Unclear | Low | Low | Unclear | Low |
| Beevi Z, et al. 2016 | High | High | High | Unclear | High | Unclear | Low |
| Beevi Z, et al. 2017 | High | High | High | Unclear | High | Unclear | Low |
| Garcia GJ, et al. 2017 | Low | Low | High | Unclear | Low | Unclear | Low |
| Novelia S, et al. 2018 | Low | Unclear | Unclear | Low | Low | Unclear | Low |
| Garcia GJ, et al. 2018 | Low | Low | High | Unclear | Low | Unclear | Low |
| Beevi Z, et al. 2019 | High | High | High | Unclear | High | Unclear | Low |
| Wan-Lin P et al. 2019 | Low | Low | Low | Unclear | Low | Unclear | Low |
| Ahmadi M, et al. 2019 | Low | Low | Unclear | Unclear | Low | Unclear | Low |
| Rajeswari S., et al. 2020 | Low | Low | Unclear | Unclear | Low | Unclear | Low |
| Zarenejad M., et al. 2020 | Low | Unclear | Unclear | Low | Low | Unclear | Low |
| Abd Elgwad FMH., et al. 2021 | Low | Unclear | Unclear | Low | Low | Unclear | Low |
| Bauer I., et al. 2021 | Low | Unclear | Unclear | Low | Low | Unclear | Low |
| Estrella-Juarez F. et al. 2022 | Low | Unclear | Unclear | Low | Low | Unclear | Low |
| Abarghoee SN. et al. 2022 | Low | Unclear | Unclear | Low | Low | Unclear | Low |
| Ghorbanneja d S, et al. 2022 | Low | Unclear | Unclear | Low | Low | Unclear | Low |
