# Supplementary material for: Effects of relaxation interventions during pregnancy on maternal mental health, and pregnancy and newborn outcomes: A systematic review and meta-analysis
Source: PLoS One. 2024 Jan 25;19(1):e0278432. doi: 10.1371/journal.pone.0278432 (PMC10810490; doi:10.1371/journal.pone.0278432)
Supplement: Attachment — Submitted filename: Response to Reviewers.docx [file pone.0278432.s003.docx]

**Journal: PLOS One**

**Response to Feedback**

***Our general response:*** *The authors are thankful to the reviewers for taking their time to read and give us important feedback/comments on our manuscript. Now we have considered the comments and have done important edits throughout the document to improve the quality and intensity of the manuscript. Below please find point-by-point response to the issues raised. We have used track change in the main document to indicate changes.*

1. Pregnant women at risk of preterm births are likely to have more anxiety, depression etc. Why were they excluded from the analysis?

***Response:*** Thank you for this comment. Now we have included those articles in to the review.

2. Although methodology section mentions to study impact in early postnatal life. Details are missing.

***Response:*** Now this is clarified that the aim of this study is to examine the effect of relaxation intervention on maternal wellbeing, pregnancy and birth and newborn outcomes. Our study does not assessed the impact during the early postnatal life.

3. This study should be included in the review: Effects of Benson Relaxation Technique and Music Therapy on the Anxiety of Primiparous Women Prior to Cesarean Section: A Randomized Controlled Trial: <https://www.hindawi.com/journals/arp/2022/9986587/>

***Response:*** Now we have added this and other articles identified when we update the searching. Thus a total of 13 articles are added now. Consequently we have revised the manuscript consistently throughout the text.

4. Since it has been more than a year since the search, I suggest you do the search again.

***Response***: Now we updated this on 26 August 2023 and made the desired revisions.

5. Some citation did not reveal in the text such as P 9.

**Response**: Now corrected
