# Supplementary material for: Effects of relaxation interventions during pregnancy on maternal mental health, and pregnancy and newborn outcomes: A systematic review and meta-analysis
Source: PLoS One. 2024 Jan 25;19(1):e0278432. doi: 10.1371/journal.pone.0278432 (PMC10810490; doi:10.1371/journal.pone.0278432)
Supplement: Attachment — Submitted filename: Response to Reviewers.docx [file pone.0278432.s004.docx]

**14/11/2023**

**To PLOS One Journal**

**Response to review feedback**

***Our general response:*** *The authors are thankful to the reviewers for taking their time to read and give us important feedback/comments on our manuscript. Now we have considered the comments and have done important edits throughout the document to improve the quality and intensity of the manuscript. Below please find point-by-point response to the issues raised. We have used track change in the main document to indicate changes.*

1. As authors keep on revising their manuscript, they must make sure the revision is reflected throughout the manuscript such that there are no anomalies in numbers (e.g., the number of studies for stress, and anxiety) and reports. For instance, findings reported in the results section were different from the Figures (e.g., Fig 1—PRISMA flow charts, Figure 2 etc). Also, for some of the Figures (Figure 2, 3), if the reference is the same, authors may differentiate them with “a” and “b” so readers may appropriately identify the exact study being referred to. For instance, Guardinoa et al. 2014a and Guardinoa et al. 2014b. If it is the same study, the same style can be used but it may be indicated beneath the figure as note “that the Guardinoa et al. 2014a and –2014b are from the same study. Please authors should make sure they rectify all these inconsistencies.

**Response:** These now are corrected on the search flow diagram (fig 1), and all other figures (fig 2-5) by avoiding inconsistencies in the number and type of studies. Findings from similar studies are noted as ‘a’ and ‘b” as recommended by reviewer.

2. Nine trials on maternal perceived stress during pregnancy using PSS (30–38), 12 trials on anxiety during pregnancy using the State-Trait Anxiety Inventory (S-TAI) (30,31,33,35,38–45), 7 trials on antenatal and postnatal depression using the Edinburgh Postnatal Depression Scale (EPDS) (31,37,41,42,44,46,47), and 6 trials reported birth weight in grams or kilograms (44,48–52)?

**Response:** this statement is now also revised and clarified in the main document as “Among the 27 RCT studies, 9 trials were on maternal perceived stress during pregnancy using PSS (35–43), 13 trials on anxiety during pregnancy using the State-Trait Anxiety Inventory (S-TAI) (35,36,38,40,43–51), 7 trials on antenatal and postnatal depression using the Edinburgh Postnatal Depression Scale (EPDS) (30,36,42,46,47,49,52), and 8 trials were on birth weight and reported weight in grams or kilograms (31,49,53–58).

3. The reviewer commented on a missing reference for a study that was added. In all, I suggest that the authors thoroughly go through and revise the manuscript to make sure there are no inconsistencies or missing data/information in the manuscript. Especially, they should go through the results section once more and update the discussion section appropriately.

***Response:*** Now missing references are included (4 studies). Moreover the result and discussion section has been thoroughly edited.

4. Some studies that were added newly were not included in the reference list. For instance, Abarghoee SN, et al. 2022

**Response**: now this and other missed studies are included.
