# Supplementary material for: Effects of relaxation interventions during pregnancy on maternal mental health, and pregnancy and newborn outcomes: A systematic review and meta-analysis
Source: PLoS One. 2024 Jan 25;19(1):e0278432. doi: 10.1371/journal.pone.0278432 (PMC10810490; doi:10.1371/journal.pone.0278432)
Supplement: Attachment — Submitted filename: Response to Reviewers.docx [file pone.0278432.s005.docx]

**24/11/2023**

**To PLOS One Journal**

**Response to review feedback**

***Our general response:*** *we are really very thankful to the editor for giving us this opportunity to correct and make thorough edits to the manuscript. Accordingly all the authors including neutral reader have gone through the manuscript and made a detail read and added the necessary edits and corrections. Moreover below we have indicated for specific corrections we have made.*

1. Going through the data once more, I realized that the data were wrongly reported in Figure 2 (stress). Please recheck the mean difference for PMR/BR. It is not -13.1 but rather -8.1, (95%CI -17.80, 1.6) and this mean difference is not significant.

**Response:** Again we thank you for this feedback. The confusion happen when we report finding from individual PMR/BR trial without reporting the finding from the overall subgroup analysis in the text. So now we have clarified and corrected this as follow **on page 10**. “In a subgroup analysis although PMR/BR as a group showed no significant association (mean difference -8.1, 95% CI: -17.8, 1.6), an individual PMR/BR trial (mean difference: -13.1; 95% CI:-15.3, -11.0) and meditation therapy (mean difference: -13.1; 95% CI:-14.0, -12.2) showed the highest effect size while music therapy as a group showed the lowest effect size (mean difference _=_ -0.8; 95% CI:-1.5, -0.1).”

2. The same issue, especially with PMR/BR can be found in anxiety figure 3. Again, although MD was -8.61 the 95%CI contains “0” which means it is not significant.

**Response:** We have also corrected and clarified this as follows **on page 11**. “In the subgroup analysis although PMR/BR trials as a group showed no association (mean difference: -8.6; 95% CI: -22.6, 5.4), individual PMR/BR trials showed the highest effect size of -15.8 (mean difference: -15.8; 95% CI: -18.3, -13.3) and lowest effect size of -1.5 (mean difference: -1.5; 95% CI: -2.9, -0.1) in reducing symptoms of anxiety.”

3. Cortical spelling errors on Trial vs Trails:

**Response:** We are very sorry for this and we have corrected them on three different sites in the main document **page 9, 11 & 12**.

4. I will entreat the authors to go through it once more or better still let another neutral person edit it before re-submission.

**Response:** Thank you for this supportive feedback. Now the paper is thoroughly read and edited by all the authors and neutral reader and made important edits as can be seen in the track changed version of the main document.
