# Supplementary material for: Effects of relaxation interventions during pregnancy on maternal mental health, and pregnancy and newborn outcomes: A systematic review and meta-analysis
Source: PLoS One. 2024 Jan 25;19(1):e0278432. doi: 10.1371/journal.pone.0278432 (PMC10810490; doi:10.1371/journal.pone.0278432)
Supplement: Attachment — Submitted filename: Response to Reviewers.docx [file pone.0278432.s006.docx]

**15/12/2023**

**To PLOS One Journal**

**Response to review feedback**

***Our response:*** *Again we are very thankful to the editor for giving us these review feedback. Accordingly we have revised and made a careful necessary corrections. Below we have indicated for specific corrections we have made.*

1. Authors should be consistent in how they report the sub-group analysis. A significant MD is what is important first and then the size of the MD and not the other way round. That is, if the MD is super high but it is not significant means nothing research-wise although noteworthy. Hence, I may suggest that authors report those MDs that are significant and then the MD sizes of those significant MDs. Then other super high non-significant MD may be mentioned as noteworthy cases.

***Response:*** *Thank you for this comment. Now we have corrected this accordingly and followed a consistent reporting for subgroup analysis in the text. Corrections are made on page 10-12.*

2. Authors may choose to mention that the MD of for instance PMR/BR is the highest but the point is that it is not significant and so, research-wise, it does not matter as it can be equated to the other non-significant sub-group analysis. The main point is to clearly discuss why you had these results in the discussion section. In other words, clearly discuss the reasons behind the high MD but non-significant results. This applies to stress and anxiety.

***Response:*** *Yes we agree and accept this critical comment. Now we have chosen to report in the text only those that are statistically significant and discussed them accordingly. Corrections are made on page 10-12 and also page 14-16.*

3. Again, if authors will be mentioning sub-group results, then they should be ready to discuss those results. The discussion should also mention the limitations including the effect of single studies for some sub-groups. This is to help consumers understand the results clearly and know the pros and cons of using your findings. As mentioned previously, this manuscript will go a long way to influence clinical decisions and so sentences should be constructed with great caution.

***Response: This*** *is also well addressed in the discussion and limitation sections. Corrections are made on page 14-16.*

4. Limitations: single studies in some subgroups analysis and their effect

***Response:*** *This is now added in the limitation section as “Because of lack of literature, some of the subgroup analysis involved only single study”. Corrections are made on page 17.*
